# Body Composition and Kinematic Analysis of the Grab Start in Youth Swimmers

**DOI:** 10.2478/hukin-2014-0057

**Published:** 2014-10-10

**Authors:** Ahmet Alptekin

**Affiliations:** 1 Pamukkale University, School of Sport Sciences and Technology, Department of Coaching Education, Turkey.

**Keywords:** swimming, grab start, kinematic analysis, somatotype, youth swimmers

## Abstract

The purposes of this study were to compare the kinematic variables in youth swimmers during the grab start between sexes and to investigate the relationship between body composition and kinematic variables of the participants. Six female (M_age_ = 13.71 ± 0.49 yrs) and seven male (M_age_ = 14.00 ± 1.07 yrs) swimmers participated in this study. All participants were required to perform grab start tests in random order (three trials by each participant), while the best attempt was analyzed. Nineteen kinematic parameters consisting of block time, flight time, flight distance, total time, total distance, horizontal and vertical displacement of the center of mass (CM) at take-off, horizontal and vertical displacement of the CM at entry, height of take-off and entry, relative height of take-off, horizontal and vertical velocity of the CM at take-off, horizontal and vertical velocity of the CM at entry, angle of take-off, angle of entry and angle of knee at block were analyzed. Out of the 19 evaluated kinematic parameters, a statistical difference between the female and male group was found only in the total distance. Therefore, both female and male groups are considered as only one group and merged after analyzing the results. Statistical analysis showed positive and negative correlations between horizontal / vertical velocity of CM at take-off and several kinematic variables (e.g. angle of entry (r_horizontal_ = −.868, p=.000 / r_vertical_ = .591, p=.02), total distance (r_horizontal_ = .594, p=.02 / r_vertical_ = .54, p=.04), and height of take-off (r_vertical_ = .888, p=.000), respectively). On the other hand, positive and negative correlations were found between somatotype components and several kinematic variables (e.g. horizontal displacement of CM at entry (r_endomorphy_ = −.626, p=.013), angle of entry (r_mesomorphy_ = −.686, p=.005 / r_ectomorphy_ = .52, p=.047), total distance (r_endomorphy_ = −.626, p=.012), and height of take-off (r_endomorphy_ = −.633, p=.011 / r_ectomorphy_ = .515, p=.05)). In conclusion, results show that in order to be successful at grab start performance, a swimmer should target to get higher horizontal velocity of CM at take-off and optimize the angle of take-off so this movement form supplies more total distance to the swimmer. Coaches should consider improving start performance and adding start training to regular training sessions. Moreover, youth male and female swimmers can participate together in the grab start training.

## Introduction

Many factors that affect start performance in swimming have been studied generally in adults (Guimaraes and Hay, 1985; Holthe and McLean, 2001; Issurin and Verbitsky, 2002; Gautier et al., 2004; Galbraith et al., 2008; Vantorre et al., 2010; Puel et al., 2012). In several investigations, the anthropometric and biomechanical characteristics in pubertal swimmers have been studied (Jürimäe et al., 2007; Latt et al., 2009; Latt et al., 2010); whereas in some of the studies, the relationship between these characteristics and swimming performance has been examined (Klentrou et al., 1991; Siders et al., 1993).

The grab start technique has been almost globally accepted as the most effective start because of its potential biomechanical advantages (Zatsiorsky et al., 1979; Blanksby et al., 2002; Chen and Tang, 2005). In competitive swimming, the aim is to get over more ground in the last amount of time; from this point of view, a swimmer must start, swim and turn as fast as possible. Even if the block time is generally too short (between 0.8% and 26.1% of the overall race time depending on the event), while comparing the other parts of swimming, this little time can be a determiner on the swimming performance (Cossor and Mason, 2001). To produce a fast entry, the take-off velocity must be high and then a streamlined position underwater should be maintained to minimize the loss of horizontal velocity. According to Cossor and Mason (2001), the start phase of the swimming should be analyzed in more detail in order to determine the sub phases within a start that the most influence the overall starting time.

Kinematic variables provide further information regarding technique for coaches. Modern tests, which are used for testing high performance athletes, need not only to be reliable, objective and valid, but also situational-specific (Dopsaj et al., 2003). On the other side, growth and maturation periods are related and both of them have an effect on physical performance. For this reason, specific body composition and body proportions are closely related with participation in a particular sport discipline. Therefore, it is believed that swimmers should start intensive swimming training before pubertal age to be successful at international level of competition (Erlandson et al., 2008).

The start in swimming, together with the other components of the competition, is very important for reaching better results. However, data regarding the kinematic parameters which affect start performance during the pubertal period is limited in youth swimmers.

The purposes of this study were to compare the kinematic variables in youth swimmers during the grab start between sexes and to investigate the relationship between body composition and kinematic variables of the participants.

## Material and Methods

In this study, the following parameters were computed: block time, flight time, flight distance, total time, total distance, horizontal -vertical displacement of center of mass (CM) at take-off, horizontal - vertical displacement of CM at entry, height of take-off, height of entry, relative height of take-off, horizontal - vertical velocity of CM at take-off, horizontal - vertical velocity of CM at entry, angle of take-off, angle of entry and angle of knee. Additionally, somatotype components were calculated from the anthropometric measurements.

### Participants

Six female (M_age_ = 13.71 ± 0.49 yrs) and seven male (M_age_ = 14.00 ± 1.07 yrs) swimmers participated in this study after giving informed consent to the experimentation. The descriptive characteristics, body composition and somatotype components of the youth swimmers are presented in Table 1.

In addition, the 100 m performance values and FINA points of the youth swimmers participating in the study are shown in Table 2.

The Pamukkale University (PAU) Ethics Committee approved all procedures and before data collection, informed consent was gained from both parents and children, respectively.

### Study design

The study was carried out at the Olympic Swimming Pool of PAU Sport Center and all participants were required to perform grab start tests in random order (three trials by each participant); the best result was used for further analysis.

### Kinematic analysis

**Figure 1:**
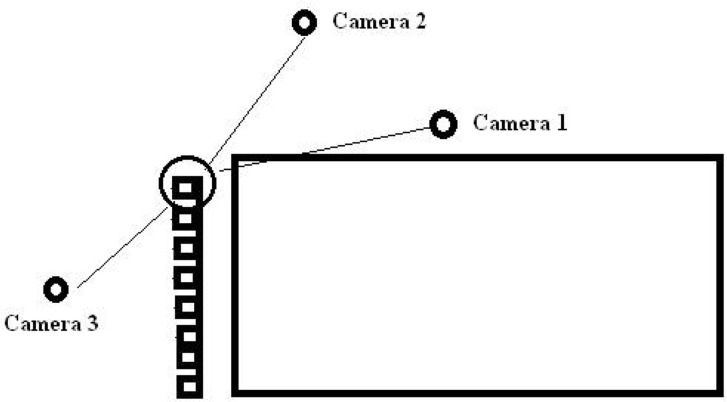
Location points of the cameras in the experimental setups.

The grab start was recorded with three high speed cameras which were set at 100 frames per second (Basler A602f-HDR GmbH, GER). The kinematic analysis of the swimmers’ grab start was performed as 3 dimensional (3D). The reflective markers, which were attached to the left side of the swimmers, were recorded with camera 1 and camera 2. On the other hand, the reflective markers, which were attached to the right side of the swimmers, were recorded with camera 1 and camera 3. Therefore, cameras were placed at different angles in the plane of motion and on the starting block. Two cameras were placed on the left side and the third camera was placed on the right side of the plane of the motion and the starting block (Figure 1); the plane of motion was calibrated vertically and horizontally by using a rigid pole with visible markings.

For digitization, reflective markers were attached to joints (5^th^ matetarsal, lateral malleolous, lateral femoral epicondyl, trochanter major, acromion, lateral epicondyl of humerus, lateral styeloid of radius) on the left and right side of the subjects in addition to their chin and forehead. Kinematic data was digitized and analyzed using SIMI 7.5 motion analysis software (SIMI Reality Motion Systems GmbH, GER). Three-dimensional marker position coordinates of all markers were computed using the direct linear transformation (DLT) method (Abdel-Aziz and Karara, 1971) by means of motion analysis software. Body segments were accepted as a rigid body. Body segment weights and center of mass location were calculated with the regression equations (Chandler et al., 1975). The body center of mass location was computed from the calculated body segment’s center of mass location (Eq. 1, 2, 3).

Eq. 1 $$X_{cg}=\frac{\sum_{i}^{n}m_{i}x_{i}}{\sum_{i}^{n}m_{i}}$$

Eq. 2 $$Y_{cg}=\frac{\sum_{i}^{n}m_{i}y_{i}}{\sum_{i}^{n}m_{i}}$$

Eq. 3 $$Z_{cg}=\frac{\sum_{i}^{n}m_{i}z_{i}}{\sum_{i}^{n}m_{i}}$$

While *X_cg_*, *Y_cg_* and *Z_cg_* are location of horizontal, sagittal and vertical axes of the body center of mass; *x*, *y* and *z* are location of horizontal, sagittal and vertical axes of each segment’s center of mass; *m* is mass of each segment and *i* is the number of segment.

The abbreviations and definitions of 19 kinematic variables that were analyzed in this study are given in Table 3.

**Figure 2:**
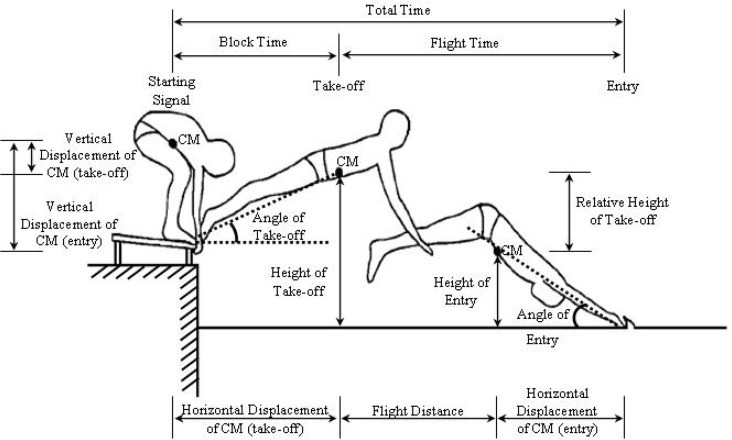
Diagrammatic presentation of selected variables (it was revised from Guimares and Hay’s article published in 1985).

Diagrammatic presentation of selected variables is shown in Figure 2.

### Anthropometric measurements

Skinfold thickness was measured two times on the right side of the body by using a skinfold caliper (10 g/mm^2^ constant pressure), (Holtain Ltd., UK). The diameters (bicondylar width of the femur and humerus) were measured with a Harpenden anthropometer (±1 mm), (Holtain Ltd., UK). The circumferences (biceps and calf) were measured with a Gullick meter (±1 mm). Anthropometric measurements were taken by a qualified person with professional experience of more than 10 years. The researcher was also a member of ISAK (Level 1). The endomorphic and mesomorphic somatotype ratings were calculated from triceps, scapula, suprailiac and medial calf sites. When the difference between two measurements was greater than 0.5 mm, additional measurements were repeated at that site until 2 of the trials were within 0.5 mm of each other. Endomorphy, mesomorphy and ectomorphy values were computed using the Heath-Carter somatotype techniques (Carter and Heath, 1990). All anthropometric measurements were taken by the same researcher. Percentage of fat was calculated by using the Slaughter et al.’s (1988) equation (Eq. 4).

Eq. 4 $$\mathrm{PBF}=1.21*(\mathrm{triceps}+\mathrm{subscapular})-.088*{\left(\mathrm{triceps}+\mathrm{subscapular}\right)}^{2}-3.4$$

### Statistical analysis

To remove noise from all raw position data of the joints it was smoothed by using a second-order Butterworth low-pass filter with a cut-off frequency of 6 Hz. All computing was performed by Matlab 5.3 software.

The t-test (Mann Whitney U) for independent samples was used to analyze the kinematic differences between the female and male groups. The Pearson’s correlation coefficient was calculated for the variables to identify a relationship between selected parameters. The level of significance was set at p<0.05. All statistical analyses were carried out using the Statistical Package for Social Science version 15.0 (SPSS, Chicago, IL, USA).

## Results

Out of 19 kinematic parameters, a statistical difference between the female and male groups was found only in the length of total distance (TD) (Table 4). Because of this reason, the TD was ignored and female and male groups were considered as one group. The differences in the group sizes and relatively low numbers decreased the statistical power and could have reduced the chance of achieving statistical significance.

Pearson Correlation results among height of take-off (HT), angle of entry (AE), total distance (TD), block time (BT) and flight distance (FD), horizontal displacement of CM at take-off (HDCMT), horizontal displacement of CM at entry (HDCME), horizontal velocity of CM at entry (HVCME) vertical velocity of CM at take-off (VVCMT), horizontal velocity of CM at take-off (HVCMT), vertical velocity of CM at entry (VVCME), angle of take-off (AT) and angle of entry (AE) are presented in Table 5.

Table 1 shows the mean values and standard deviations (mean±SD) for the descriptive characteristics, body composition and anthropometric measurements of the youth male and female swimmers. Female swimmers had higher values for % fat and endomorphy, but male swimmers had higher values for BMI, mesomorphy and ectomorphy. The results of the dependent Mann Whitney U test indicated that there was a significant difference between female and male groups only in endomorphy and % fat values.

Table 6 contains the computed correlation of anthropometric measurements (endomorphy, mesomorphy, ectomorphy, BMI, % fat) with total distance (TD), horizontal displacement of CM at entry (HDCME), height of take-off (HT), relative height of take-off (RHT), vertical velocity of CM at take-off (VVCMT), horizontal velocity of CM at entry (HVCME), vertical velocity of CM at entry (VVCME), and angle of entry (AE). Correlation analysis demonstrated that endomorph and body fat had a negative correlation with TD (r_endomorph_ = −.626, p=.012 / r_bodyfat_ = −.605, p=.017), HDCME (r_endomorph_ = −626, p=.013 / r_bodyfat_ = −.593, p=.02), HT (r_endomorph_ = −.633, p=.011 / r_bodyfat_ = −.600, p=.018), VVCMT (r_endomorph_ = −.636, p=.011 / r_bodyfat_ = −.609, p=.016), but, contrary to these results, VVCME had a positive correlation with endomorphy and body fat (r_endomorph_ = .689, p=.004 / r_bodyfat_ = .637, p=.011). As shown in Table 6, ectomorphy had a positive relationship with HT (r_ectomorphy_ = .515, p=.05), RHT (r_ectomorphy_ = .564, p=.029) and AE (r_ectomorphy_ = .520, p=.047), but was negatively related to VVCME (r_ectomorphy_ = −.517, p=.049). Mesomorphy had a negative relationship with AE (r_mesomorphy_ = −.686, p=.005) and a positive relationship with HVCME (r_mesomorphy_ = .625, p=.013).

## Discussion

The importance of the start can be clearly confirmed by the fact that in the 2012 Summer Olympic Games held in London, only 0.22 s separated the first and the third place in the men's 50 meter freestyle and in the women's 100 meter freestyle the first and the third places were separated by 0.44 s (http://www.olympic.org/olympic-results/london-2012/swimming). Generally, the swimmer’s target should be the mixture of quickness off the block and a large forward speed that allows for the best results. The findings to date have shown that, regardless of the swim start choice, the swimmers’ goal is to react quickly to the starting signal, leave the block rapidly generating as much horizontal velocity as possible, gain maximal flight distance while using an optimal angle of entry, and maintain a streamline position that will minimize the loss of horizontal velocity related with the drag on water entry (Guimaraes and Hay, 1985; Issurin and Verbitsky, 2002; Maglischo, 2003; Galbraith et al., 2008). Numerous studies have evaluated and compared start performances with mixed results (Breed and Young, 2003; Galbraith et al., 2008; Jorgic et al., 2010) and most important variables, which affect the swimmers performance have been indicated as block time and the take-off angle (Hay, 1993; Maglischo, 2003). Moreover, a study of 50 m and 100 m freestyle events examining the relationship between the final performance (total time and total records) and block times with the two different starting platforms (old and new) indicated that the block time for the men’s 50 m freestyle was positively correlated with the final time in the semi-finalist, medalist groups and overall sample, and the block time for the men’s 100 m freestyle showed correlations with the final time between the finalists and overall sample. For women, similar relationships (between the block time and final time) were observed between the finalist, semi-finalist groups and overall sample for the 50 m freestyle event. In the 100 m freestyle event in women, the correlations were observed with the semi-finalists and overall sample (Garcia-Hermoso et al., 2013). From this point of view, in this study, we focused on the swimmer’s movements on the block and in the air while performing the grab start. The analysis consisted of the parameters which affect grab start performance according to the study of Guimaraes and Hay (1985). They concluded that, to obtain a faster start, swimmers should move towards the CM as fast as possible in the forward direction when the feet were still on the block, and they also showed that velocity and distance variables were significantly correlated with starting time on the block. In addition to this, Maglischo (2003), based on several studies, considered that in the grab start, the angle of take-off was around 300–400. Unlike the Maglischo’s study, Heusner (1959) indicated that the angle of take-off varied between 50–220 and the optimum angle of take-off under normal circumstances was 130. As in the Heusner’s study, the angle of take-off varied between 3 and 170 (Hay, 1978). One the other hand, one of the key components of dive performance in previous studies was increasing flight distance (Breed and Young, 2003; Galbraith et al., 2008).

In the current study, swimmers achieved significantly greater flight distance when height of take-off was higher. A positive correlation was found between flight distance and height of take-off indicating that more flight distance was associated with a higher take-off height (r = .76, p=.001), and flight distance and total distance (r = .786, p=.001). On the other hand, a negative correlation was found between horizontal displacement of CM at entry and the angle of entry which indicates more distance horizontally means a smaller angle of entry (r = −.577, p=.02), but a positive correlation was found between horizontal displacement of CM at entry and total distance, which was an expected result (r = .721, p=.002). Additionally, a positive correlation was found between horizontal velocity of CM at entry and the angle of entry (r = .591, p=.02), and horizontal velocity of CM at entry and total distance (r = .698, p=.038). Therefore, greater forward velocity of CM was associated with a higher entry angle and more distance at entry totally. A positive correlation was found between vertical velocity of CM at take-off and height of take-off (r =.888, p=.000), vertical velocity of CM at take-off and the angle of entry (r = .591, p=.02), vertical velocity of CM at take-off and total distance (r = .54, p=.038). Hence, the higher velocity the swimmer has vertically, the further he/she will gain height of take-off and the angle of entry and trajectory before entering the water. In this study, a negative correlation was found between horizontal velocity of CM at take-off and the angle of entry indicating a negative effect of the angle of entry on horizontal velocity (r = −.868, p=.000), but a positive correlation was revealed between horizontal velocity of CM at take-off and total distance (r = .594, p=.02). Therefore, the higher forward position of CM was associated with greater horizontal velocity of CM at take-off and a lower angle of entry. On the other hand, a negative correlation was found in vertical velocity of CM at entry, height of take-off (r = −.788, p=.000), the angle of entry (r = −.636, p=.011) and total distance (r = −.565, p=.028), indicating that more forward position of CM at entry is associated with a vertically slower velocity at entry. A positive correlation was found between the angle of entry and height of take-off (r = .677, p=.006). The movement form, from take-off to the entry in water, is very similar to projectile motion in physics. In this motion form, angles of take-off and entry are the same when the air resistance is disregarded. Based on the take-off angle and velocity of projectile, it gets higher or lower distance horizontally/vertically, so the higher angle of take-off means higher distance vertically/horizontally. From this point of view, under the same conditions, when the angle of entry is high, the swimmer gets higher height of take-off and accordingly gets less flight distance which means less total distance.

In addition, several researchers have investigated the anthropometric characteristics of swimmers and examined the relationship between these characteristics and the swimming performance between sexes (Chatard et al., 1990; Fortes and Castro, 2002; Jürimäe et al., 2007; Seifert et al., 2007; Latt et al., 2009). Some of them found that male swimmers were generally balanced mesomorphs or ectomorphs but unlike male swimmers, female swimmers showed a difference in their somatotype rating. They were generally higher in endomorphy and lower in mesomorphy than male swimmers (Zuniga et al., 2011). Our findings support these previous findings. In the current study, female swimmers had higher endomorph and body fat percentage values than male swimmers, and male swimmers were balanced mesomorphs and ectomorphs, had lower body fat percentage values but higher BMI. When we checked the correlation between somatotype measurements and chosen kinematic parameters, it can be concluded that higher endomorph values negatively affected total distance, horizontal displacement of CM at entry, height of take-off, relative height of take-off, vertical velocity of CM at take-off, horizontal velocity of CM at entry and the angle of entry which means male swimmers had better results at the grab start in this study group. The only meaningful difference between female and male group was total distance. This can be explained by the higher mesomorph and ectomorph values in males than females and also because of the sex difference which can be explained by the differences in the male and female puberty period.

In summary, the results of the present study indicate that the only differences between female and male swimmers were for measures of %fat, endomorph and total distance measurements. Further studies can be carried out to examine sex differences by increasing the number of the subjects and measuring ground reaction forces and muscle activity during the grab start in particular age group in both sexes.

In conclusion, the results of this study indicate that to be successful at the grab start, swimmers should target to get higher horizontal velocity of the center of mass at take-off and optimize the angle of take-off so this movement form provides the swimmer with more total distance.

## Figures and Tables

**Table 1:** Descriptive characteristics, body composition and somatotype components of the youth swimmers (mean ± SD).

	**Female group (n=6) Mean (±SD)**	**Male group (n=7) Mean (±SD)**	**All group (n=13) Mean (±SD)**
**Age (yrs)**	13.71 (0.49)	14.00 (1.07)	13.87 (0.83)
**Body height (cm)**	157.14 (3.98)	164.00 (5.53)	160.80 (5.88)
**Body mass (kg)**	49.93 (8.04)	58.91 (9.32)	54.72 (9.63)
**Training experience (yrs)**	4.71 (1.11)	6.13 (2.23)	5.47 (1.88)
**BMI**	20.62 (2.65)	21.75 (4.44)	21.23 (3.62)
% fat^*^	23.07 (5.74)	13.88 (4.46)	18.12 (6.82)
Endomorphy^*^	4.47 (1.24)	2.56 (1.09)	3.44 (1.49)
**Mesomorphy**	2.83 (0.72)	3.12 (1.95)	2.99 (1.46)
**Ectomorphy**	2.58 (1.18)	2.87 (2.00)	2.74 (1.61)

% fat = body fat percentage.

* Significant difference (p<0.05) between female and male groups.

**Table 2:** The performance level of the youth swimmers

		**Freestyle**		**Backstroke**		**Breaststroke**		**Butterfly**		**Medley**	

**Sexes**	**Subjects number**	**100 m**	**FINA points**	**100 m**	**FINA points**	**100 m**	**FINA points**	**100 m**	**FINA points**	**100 m**	**FINA points**
**Female**	**1**	01:09.93	412	01:27.82	289	01:26.29	416	01:27.89	259	-	-
	**2**	01:09.97	412	01:25.25	316	01:25.46	428	01:17.36	380	01:16.54	429
	**3**	-	-	01:11.56	535	-	-	01:12.41	464	-	-
	**4**	01:23.19	245	-	-	02:00.44	153	01:48.56	137	01:36.40	214
	**5**	01:17.98	297	-	-	01:37.23	291	-	-	-	-
	**6**	01:10.04	410	01:12.04	525	01:44.71	233	01:35.46	202	-	-
**Male**	**1**	-	-	01:07.60	453	01:15.71	463	01:03.54	482	01:02.69	530
	**2**	-	-	-	-	-	-	01:07.99	393	-	-
	**3**	01:21.60	189	01:18.45	290	01:41.51	192	01:29.48	172	-	-
	**4**	01:02.20	428	-	-	01:13.00	516	-	-	-	-
	**5**	01:06.30	354	01:11.36	385	01:26.35	312	01:07.60	400	01:10.02	380
	**6**	01:02.53	422	01:23.59	239	01:12.01	538	01:20.38	238	01:09.94	382
	**7**	01:09.40	308	-	-	01:24.49	333	01:30.87	164	-	-

**Table 3:** The analyzed variables and its abbreviations and definitions.

**Analyzed Variables**	**Abbreviations**	**Definitions**
**Block time (s)**	BT	The time from the starting signal until take-off from the block
**Flight time (s)**	FT	The time the swimmer spent in the air
**Flight distance (m)**	FD	The distance of the CM from the last contact of the foot from the starting block to the first contact of the hands with the water at the exact position
**Total time (s)**	TT	The time the swimmer spent from the starting signal until entry
**Total distance (m)**	TD	The distance the swimmer spent from the starting signal until entry
**Horizontal displacement of the CM at take-off (m)**	HDCMT	The horizontal displacement starting from the CM on the block to the last contact of the feet from the starting block
**Vertical displacement of CM at take-off (m)**	VDCMT	The vertical displacement starting from the CM on the block to the last contact of the feet from the starting block
**Horizontal displacement of the CM at entry (m)**	HDCME	The horizontal distance starting from the CM to the first contact of the hands with the water at the exact position
**Vertical displacement of the CM at entry (m)**	VDCME	The distance between the first contact of the hands with the water and the CM at the exact position
**Height of take-off (m)**	HT	Determined by the height of the CM at take-off
**Height of entry (m)**	HE	Determined by the height of the CM at entry
**Relative height of take-off (m)**	RHT	Determined as the difference between the height of the CM at take-off and the height of the CM at entry
**Horizontal velocity of the CM at take-off (m/s)**	HVCMT	The horizontal velocity of the CM at the moment of the last contact of the foot with the starting block
**Vertical velocity of the CM at take-off (m/s)**	VVCMT	The vertical velocity of the CM at the moment of the last contact of the foot with the starting block
**Horizontal velocity of the CM at entry (m/s)**	HVCME	The horizontal velocity of the CM at the moment of the first contact of the hands with water
**Vertical velocity of the CM at entry (m/s)**	VVCME	The vertical velocity of the CM at the moment of the first contact of the hands with water
**Angle of take-off (degree)**	AT	An angle between CM and the horizontal line which connects the body at the moment of the last contact of the foot with the starting block
**Angle of entry (degree)**	AE	An angle between CM and the horizontal line which connects the body at the moment of the first contact of the hands with the water
**Angle of knee at block (degree)**	AKB	Knee joint angle of at the moment of the last contact of the foot with the starting block

CM: Center of mass

**Table 4:** Means and standard deviations of kinematic variables were analyzed (Mean ± SD)

	**Male (n = 7)**	**Female (n = 6)**	**Group (n = 13)**
	
**Block time (s)**	0.68 (0.08)	0.67 (0.08)	0.68 (0.07)
**Flight time (s)**	0.37 (0.08)	0.33 (0.07)	0.35 (0.07)
**Total time (s)**	1.05 (0.09)	1.00 (0.09)	1.03 (0.09)
**Total distance (m)**	2.89 (0.18)^*^	2.51 (0.35)	2.71 (0.33)
**Horizontal displacement of CM at take-off (m)**	0.99 (0.10)	0.87 (0.13)	0.93 (0.13)
**Horizontal displacement of CM at entry (m)**	0.81 (0.08)	0.77 (0.13)	0.79 (0.10)
**Flight distance (m)**	1.09 (0.21)	0.81 (0.23)	0.96 (0.26)
**Total vertical displacement of CM (m)**	0.54 (0.09)	0.58 (0.06)	0.56 (0.08)
**Height of take-off (m)**	1.20 (0.09)	1.08 (0.11)	1.14 (0.11)
**Height of entry (m)**	0.71 (0.09)	0.63 (0.11)	0.67 (0.09)
**Relative height of entry (m)**	0.48 (0.09)	0.45 (0.09)	0.47 (0.09)
**Horizontal velocity of the CM at take-off (m/s)**	3.32 (0.23)	2.91 (0.51)	3.13 (0.42)
**Vertical velocity of the CM at take-off (m/s)**	0.12 (0.64)	0.64 (0.48)	0.37 (0.61)
**Horizontal velocity of the CM at entry (m/s)**	0.71 (0.07)	0.64 (0.10)	0.67 (0.09)
**Vertical velocity of the CM at entry (m/s)**	0.67 (6.05)	0.62 (0.07)	0.65 (0.06)
**Angle of take-off (deg)**	8.75 (6.10)	13.37 (7.75)	10.91 (7.07)
**Angle of entry (deg)**	43.69 (5.10)	44.46 (6.09)	44.05 (5.39)
**Angle of knee at block (deg)**	161.30 (12.73)	159.66 (24.70)	160.54 (18.54)

* Significant difference with the male group (U=9.0, p=0.028<0.05).

CM: Center of mass

**Table 5:** Correlation between the selected variables (from the point of duration, velocity and angle)

**n = 13**		**Height of take-off (m)**	**Angle of entry (deg)**	**Total distance (m)**	**Block time (s)**
**Flight distance (m)**	r	0.76^**^	0.268	0.786^**^	−0.096
	p	0.001	0.334	0.001	0.734
**Horizontal displacement of CM at take-off (m)**	r	−0.137	−0.46	0.461	0.174
	p	0.626	0.084	0.084	0.536
**Horizontal displacement of the CM at entry (m)**	r	−0.124	−0.577^*^	0.721^*^	0.134
	p	0.66	0.024	0.002	0.633
**Horizontal velocity of the CM at entry (m/s)**	r	−0.035	0.591^*^	0.698^*^	−0.063
	p	0.9	0.02	0.04	0.825
**Vertical velocity of the CM at take-off (m/s)**	r	0.888^**^	0.591^*^	0.54^*^	−0.186
	p	0.000	0.02	0.038	0.506
**Horizontal velocity of the CM at take-off (m/s)**	r	−0.348	−0.868^**^	0.594^*^	0.27
	p	0.204	0.000	0.02	0.33
**Vertical velocity of the CM at entry (m/s)**	r	−0.788^**^	−0.636^*^	−0.565^*^	0.231
	p	0.000	0.011	0.028	0.408
**Angle of take-off (deg)**	r	−0.338	−0.089	−0.484	0.294
	p	0.217	0.753	0.67	0.288
**Angle of entry (deg)**	r	0.677^*^		−0.192	−0.334
	p	0.006		0.494	0.224

CM: Center of mass

**Table 6:** Correlation coefficients of chosen kinematic parameters and somatotype characteristics in youth swimmers

**n = 13**		**Endomorphy**	**Mesomorphy**	**Ectomorphy**	**BMI**	**Body Fat**
**Total distance (m)**	r	−.626^*^	.003	.251	.037	−.605^*^
	p	0.012	.991	.366	.897	.017
**Horizontal displacement of the CM at entry (m)**	r	−.626^*^	−.244	.386	−.144	−.593^*^
	p	.013	.381	.156	.608	.020
**Height of take-off (m)**	r	−.633^*^	−.501	.515^*^	−.266	−.600^*^
	p	.011	.057	.050	.337	.018
**Relative height of take-off (m)**	r	−.359	−.367	.564^*^	−.323	−.290
	p	.189	.178	.029	.240	.295
**Vertical velocity of the CM at take-off (m/s)**	r	−.636^*^	−.445	.494	−.306	−.609^*^
	p	.011	.096	.061	.268	.016
**Horizontal velocity of the CM at entry (m/s)**	r	−.063	.625^*^	−.357	.494	−.086
	p	.825	.013	.191	.061	.761
**Vertical velocity of the CM at entry (m/s)**	r	.689^*^	.439	−.517^*^	.280	.637^*^
	p	.004	.101	.049	.312	.011
**Angle of entry (deg)**	r	−.278	−.686^**^	.520^*^	−.495	−.234
	p	.316	.005	.047	.061	.402

CM: Center of mass
